# A validity study of self-reported daily texting frequency, cell phone characteristics, and texting styles among young adults

**DOI:** 10.1186/s13104-015-1090-3

**Published:** 2015-04-02

**Authors:** Judith E Gold, Kimberly J Rauscher, Motao Zhu

**Affiliations:** Department of Public Health, Temple University, 1301 Cecil B. Moore Ave., Philadelphia, PA USA; West Virginia University Injury Control Research Center, PO Box 9151, Morgantown, WV USA; Centre for Musculoskeletal Research, University of Gävle, Gävle, Sweden; Department of Occupational and Environmental Health Sciences, West Virginia University School of Public Health, PO Box 9190, Morgantown, WV USA; Department of Epidemiology, West Virginia University School of Public Health, PO Box 9190, Morgantown, WV USA

**Keywords:** Young adults, College students, Cell phones, Text messaging, Validity of self-reports, Short message service (SMS)

## Abstract

**Background:**

Texting is associated with adverse health effects including musculoskeletal disorders, sleep disturbances, and traffic crashes. Many studies have relied on self-reported texting frequency, yet the validity of self-reports is unknown. Our objective was to provide some of the first data on the validity of self-reported texting frequency, cell phone characteristics including input device (e.g. touchscreen), key configuration (e.g., QWERTY), and texting styles including phone orientation (e.g., horizontal) and hands holding the phone while texting.

**Methods:**

Data were collected using a self-administered questionnaire and observation of a texting task among college students ages 18 to 24. To gauge agreement between self-reported and phone bill-derived categorical number of daily text messages sent, we calculated percent of agreement, Spearman correlation coefficient, and a linear weighted kappa statistic. For agreement between self-reported and observed cell phone characteristics and texting styles we calculated percentages of agreement. We used chi-square tests to detect significant differences (α = 0.05) by gender and study protocol.

**Results:**

There were 106 participants; 87 of which had complete data for texting frequency analyses. Among these 87, there was 26% (95% CI: 21–31) agreement between self-reported and phone bill-derived number of daily text messages sent with a Spearman’s rho of 0.48 and a weighted kappa of 0.17 (95% CI: 0.06-0.27). Among those who did not accurately report the number of daily texts sent, 81% overestimated this number. Among the full sample (n = 106), there was high agreement between self-reported and observed texting input device (96%, 95% CI: 91–99), key configuration (89%, 95% CI: 81–94), and phone orientation while texting (93%, 95% CI: 86–97). No differences were found by gender or study protocol among any items.

**Conclusions:**

While young adults correctly reported their cell phone’s characteristics and phone orientation while texting, most incorrectly estimated the number of daily text messages they sent. This suggests that while self-reported texting frequency may be useful for studies where relative ordering is adequate, it should not be used in epidemiologic studies to identify a risk threshold. For these studies, it is recommended that a less biased measure, such as a cell phone bill, be utilized.

## Background

Text messaging, which involves sending short messages of no more than 160 characters via a mobile device, is widespread, particularly among young adults. According to the latest data from the Pew Research Center’s Internet and American Life Project, 97% of young adult cell phone users, ages 18 to 24, engage in text messaging on their cell phones at a rate of nearly 110 messages per day or 3,200 per month [1]. Studies have examined the adverse health effects of texting and have shown that sleep disturbances [2-4], musculoskeletal disorders (MSDs) [5,6], traffic crashes, [7] sedentary behaviors [8], internet addiction [9], and developmental issues [10] may be associated with duration and/or frequency of texting. Many of these studies have relied on participants’ self-reported assessments of their texting exposures.

Unlike the fairly substantial published literature that has examined the validity of self-reported duration and frequency of cell phone calls [11-17], we are aware of only two studies that have examined the validity of self-reported texting frequency in comparison to phone company-generated data [18,19]. Self-reports of texting frequency are subject to recall bias and, potentially, social pressures to over-report/under-report. In contrast, phone company-generated data including billing records are free from such bias. One study determined the validity of self-reports among young adolescents (mean age of 12.3 years) and found overestimation in lower volume texters and underestimation in higher volume texters [18]. The other study, which surveyed a nationally representative sample in Norway, found overestimation in self-reported texting frequency as compared with a phone bill [19]. However, the average self-reported daily texting frequency in this study was only 6.2. The generalizability of the results of these studies to young adults who are prolific texters is unknown.

Validity of self-reported texting frequency has implications for current and future epidemiologic studies of text messaging and related health outcomes as 18- to-24-year-olds make up the second largest proportion of texters after the 13-to-17 year old age group [20]. To address this gap, we conducted the present study to investigate agreement between self-reported texting frequency and phone-company billing records among a sample of college students.

Awkward postures, force, and repetition have been found to be associated with the risk of MSDs [21,22]. Texting styles (which hands used to hold the phone and the phone’s orientation while texting) and cell phone characteristics (type of keying input device and key configuration) may influence joint postures, muscle force intensity and frequency of muscle activation while interacting with the phone [23]. Hence, it was also of interest to determine the correspondence of these self-reported factors with those observed. Given the sparse literature on the validity of self-reported texting frequency, we turned to studies of keyboard use to inform our hypotheses since texting on cell phones also involves the use of keyboards (whether physical or virtual). These studies have found that office workers tend to overestimate the amount of time they spend per day using a keyboard [24-26]. Therefore, we hypothesized that texters would similarly overestimate the amount of texts they send daily. Because females somewhat overestimated their computer usage in comparison with males in one study [25], we also examined the effect of gender on self-reported texting frequency validity. We further hypothesized that there would be high agreement between self-reported cell phone characteristics and observed texting styles (e.g., phone orientation while texting, hands used to hold the phone) due to the objective nature of these phenomena that require little subject recall and interpretation.

The study objectives were to determine: 1) the validity of self-reported number of text messages sent per day as compared with the number of daily texts sent as ascertained through a cell phone bill; and 2) agreement between observed and self-reported cell phone characteristics and texting styles.

## Methods

Our study design involved both a self-administered questionnaire and observation of participants with a sample of college students from West Virginia University, all of whom provided informed consent prior to participation. The study was conducted in August of 2012. The Institutional Review Board of West Virginia University approved this study.

### Recruitment & eligibility screening

Information on the study including its objective of examining the cell phone exposures of college students, eligibility criteria, and the date, time and place of the survey was sent in an email to all WVU undergraduates. The email also stated that in order to participate in the study students had to have their cell phones with them and they needed to bring a copy of their cell phone bill or know their log-in information so they could access their bill online. On the day of the survey, participants were passively recruited at a table set up in the student center of WVU. Students who approached the table were given a fact sheet about the study, told that they would be given $10 in cash for taking part, and then asked if they wanted to participate. Those who were interested were then asked a series of eligibility questions by one of the three members of the data collection team. To be eligible, students had to: 1) be between the ages of 18 and 24; 2) use a cell phone to send text messages; 3) have their cell phone with them; and 4) have either a copy of their most recent cell phone bill or have their log-in information to access their cell phone account on-line. Eligible participants were then subjected to one of two study protocols described below.

### Data collection

#### Study protocols

Participants were subjected to either questionnaire first and observed texting task second (Protocol A; n = 52) or observed texting task first and questionnaire second (Protocol B; n = 54). We did this to determine whether performing the texting task first had an impact on participants’ questionnaire responses. For Protocol A, members of the study team first determined whether the participant had their paper bill or their log-in information. If they had a bill, the team member collected the bill, recorded the participant’s cell phone number on an observation form, and handed the participant a questionnaire. Participants were instructed to return the completed questionnaire to the team member. While participants were completing the questionnaire, the study team member extracted from the cell phone bill, the start and end date of the billing period and the number of text messages sent from the participant’s cell number and recorded it on the observation form.

When the participant returned the completed questionnaire, the team member returned the phone bill and then asked the participant to retrieve his/her cell phone and perform a simple texting task. Participants were instructed to manually text (use of the voice command function was not allowed) the phrase “I’m going to the library at 5:30 do you want to meet me there?” While the participant was texting, the study team member observed the participant and recorded the following information on the observation form: the hands participants’ used to hold their phone, whether they held their phone horizontally or vertically while inputting text, whether they used a keyboard or touch screen to input text, and whether a 12-key, 20-key or QWERTY key configuration was used input text.

If a participant did not have their bill but had their log-in information, the last step in the protocol was to have the participant use one of four available lap-tops and log-in to their cell phone account on-line. Once the participant was able to pull up his/her bill on the computer, s/he alerted a study team member who then recorded the pertinent information from the on-line bill on the observation form. For Protocol B, the same procedure was followed, however, the observed texting task was the first step in the protocol and the questionnaire was completed after that.

#### Questionnaire data

A self-administered questionnaire consisting of 13 questions was used to capture participants’ estimates of their daily texting frequency, texting style, characteristics of their cell phones, and demographics. The questionnaire was a shortened version of one used in a pilot survey of adolescents and young adults (n = 220) conducted by a member of the study team (Gold, unpublished). To collect number of daily texts sent, we asked participants, “On an average day, how many text messages do you send using your cell phone?” and asked them to choose one response from the following options: 10 or fewer, 11–20, 21–50, 51–100, 101–200, 201–300, 301–500, more than 500. These categories were developed based on the answer to an open-ended question on frequency of daily texts sent asked in the pilot survey.

With regard to cell phone characteristics we asked participants to tell us the key configuration of their phone (i.e., 12-key, 20-key or QWERTY) and whether they used a keyboard (i.e., physical keys) or a touch screen (i.e., virtual keys) to input text. To determine texting styles, we asked participants to indicate which hands they used to input text and provided check box options with instructions to choose *all that apply* (“left,” “right,” “neither-put phone on a surface”). If participants checked both “left” and “right” hands, this was coded as using “both hands.” Participants were also asked whether they “most often” hold their phone horizontally or vertically (i.e., phone orientation) when they input text. Lastly, we asked how often they use the voice command function on their phones to send text messages. Response options were never, rarely, often, always.

### Variable construction

To measure phone-bill derived number of daily text messages sent, we divided the total number of texts sent during the billing period by the number of days in the billing period as shown on the cell phone bill. For participants whose phone bill did not distinguish between sent versus received texts (n = 25), we divided the total number of text messages listed on the bill in half^a^. The number of phone bill-derived daily text messages sent was then coded into the same categories as those on the questionnaire.

To measure agreement between self-reported and observed cell phone characteristics and texting styles, we created a dummy variable to indicate whether participants’ survey responses matched those observed for each of these items.

### Statistical analysis

Of the 106 participants in the study, 19 either did not have information on their cell phone bills regarding text messages or were unable to access their bills online due to technical problems. These participants were therefore excluded from the analyses examining agreement between self-reported and phone bill-derived daily texts (n = 87). All 106 were included in the analyses of phone characteristics and texting styles.

To gauge agreement between self-reported and phone bill-derived categorical number of daily text messages sent we calculated the percent of agreement and the Spearman correlation coefficient. For comparability with previous analyses of cell phone use, we also used a linear weighted kappa statistic as another measure of agreement. For the kappa, weights for categories with exact agreement were set to 1, categories adjacent to exact agreement to 0.5, and all others to 0. Lastly, we calculated the maximum attainable kappa (kmax^b^) as suggested by Dunn [27].

To ascertain agreement between self-reported and observed cell phone characteristics and texting styles we calculated percentages of agreement for each respective item measured. We also examined differences by gender and protocol. For texting frequency, we calculated the percent of agreement and the kappa statistic. For texting styles and cell phone characteristics, we determined the percent of agreement. The chi-square test was utilized to detect significant differences in texting frequency, cell phone characteristics and texting styles (α = 0.05). Statistical analyses were conducted using STATA 11 (StataCorp, College Station, TX) and MATLAB (The Mathworks, Natick, MA).

## Results

### Sample characteristics

Among the full sample (n = 106), participants ranged in age from 18 to 24 years. Ninety-seven percent was between the ages of 18 and 20 with a mean age of 19.6 (SD = 1.7). Fifty-two percent of the sample was female and the majority (78%) was white. Nearly all participants (95%) reported that they “rarely” or “never” used the voice function on their phone to send text messages indicating that they were manual texters.

### Agreement between self-reported and phone bill-derived number of daily text messages sent

According to participants’ phone bills, the average number of text messages sent per day was 57.7 (SD = 59.2). Table 1 shows the distribution of self-reported and phone bill-derived number of daily texts sent (n = 87). There was 26% (95% CI: 21–31) agreement between self-reported and phone bill-derived categorical number of daily text messages sent. Eighty-one percent of participants who did not accurately report the categorical number of daily texts sent overestimated this number. The tendency toward overestimation, which is indicated by the bolded numbers in Table 1, was apparent for each texting frequency category. The low level of agreement is also demonstrated by a weighted kappa of 0.17 (95% CI: 0.06–0.27). The maximum attainable kappa (kmax) for this dataset was 0.60, so the kappa achieved was 28% of kmax. Spearman rank correlation coefficient for the self-reported and phone-bill derived data was =0.49 (p < 0.0001).

**Table 1:** **Distribution of self-reported and phone bill-derived number of daily text messages sent among young adults ages 18–24 (n = 87)**

**Self-reported number**	**Phone bill-derived number**								
	**<=10**	**11–20**	**21–50**	**51–100**	**101–200**	**201–300**	**301–500**	**>500**	**Total**
<= 10	2*	0	2	0	0	0	0	0	4
11 – 20	**2**	4	1	1	0	0	0	0	9
21 – 50	**5**	**3**	**6**	6	1	0	0	0	21
51 – 100	**3**	**0**	**8**	6	0	0	0	0	17
101 – 200	**0**	**3**	**3**	**5**	4	1	0	0	16
201 – 300	**2**	**1**	**1**	**4**	**2**	1	0	0	11
301 – 500	**0**	**1**	**0**	**4**	**1**	**1**	0	0	7
>500	**0**	**0**	**0**	**1**	**1**	**1**	0	0	3
**Total**	14	12	21	27	9	4	0	0	87

*numbers in table are counts.

Note: Bolded values reflect over-estimation of the self-reported number.

For males (n = 43) there was 23% agreement (95% CI: 12–39) and for females (n = 43) there was 28% agreement (95% CI: 15–44), with no statistical difference in percent of agreement between genders [*χ*^2^ = 0.244, *p* = 0.621]. The weighted κ for males was 0.15 (95% CI: 0.001–0.30) and for females it was 0.15 (95% CI: 0.01-0.29).

For Protocol A (n = 41) there was 22% agreement (95% CI: 11–38) and for Protocol B (n = 46) there was 30% agreement (95% CI: 18–46), with no statistical difference in percent of agreement between study protocols [*χ*^2^ = 0.802, p = 0.370]. The weighted κ for Protocol A was 0.13 (95% CI: 0.00–0.29) and for Protocol B it was 0.19 (95% CI: 0.03–0.35).

### Agreement between self-reported and observed cell phone characteristics and texting styles

Table 2 shows the percent agreement on phone characteristic and texting style items for the sample as a whole and by gender and study protocol. With the exception of hands used to hold the phone (37%), there were high levels of agreement (≥89%) on each of these items. Eighty percent of those who inaccurately reported the hands they used to hold their phone while texting were observed using both hands yet they reported using only one hand. No statistically significant differences were detected by gender or study protocol.

**Table 2:** **Agreement between self-reported and observed cell phone characteristics and texting styles among young adults ages 18–24, by gender and study protocol**

	**Percent (95% CI)**				
	**All**	Gender*		Study protocol*	
		**(n = 105)**		**(n = 106)**	
		**Male**	**Female**	**A** ^**a**^	**B** ^**b**^
**Cell phone characteristics**					
Input device match^^^	96	98	95	98	95
	(91, 99)	(89, 100)	(85, 99)	(90, 100)	(85, 99)
		*χ* ^2^ = 0.853, *p* = 0.356		*χ* ^2^ = 0.963, *p* = 0.327	
Key configuration match^^^	89	88	89	85	93
	(81, 94)	(75, 95)	(77, 96)	(71, 93)	(81, 98)
		*χ* ^2^ = 0.047, *p* = 0.828		*χ* ^2^ = 1.599, *p* = 0.206	
**Texting styles**					
Phone orientation match^^^	93	92	95	89	96
	(86, 97)	(81, 98)	(85, 99)	(77, 96)	(87, 100)
		*χ* ^2^ = 0.273, *p* = 0.602		*χ* ^2^ = 2.330, *p* = 0.127	
Hands holding phone match^^^	37	38	36	36	39
	(29, 48)	(25, 54)	(24, 50)	(24, 51)	(26, 54)
		*χ* ^2^ = 0.064, *p* = 0.800		*χ* ^2^ = 0.106, *p* = 0.745	

^*^Degrees of freedom = 1.

^^^Observed characteristic matched the self-reported response from the questionnaire.

^a^Self-administered questionnaire first, then observation of texting task.

^b^Observation of texting task first, then self-administered questionnaire.

## Discussion

In this study of the validity of self-reported daily texting frequency among young adults 18 to 24 years of age, few individuals accurately estimated the number of text messages they sent. Much as computer users in office settings overestimate their keyboard usage for typing [24-26], we found young adults also overestimate their keyboard usage for texting on cell phones. Average overestimation magnitudes in computer keyboard users ranged from 2.2–4.2 [24-26]. In the current study, it was not possible to precisely compute the overestimation magnitude because the self-report of daily texts sent variable was categorical.

We found young adults consistently overestimated this number in comparison to that derived from their cell phone bills regardless of the volume of texts sent. This is in contrast to a prior study which found that low volume texters overestimated and high volume texters underestimated their texting frequency as compared with phone company information (17). Inclusion criteria for that study required participants to be on a texting plan. It is unknown how the presence of a texting plan influenced their results. Unlike that study, we did not inquire about the texting plans of our subjects. Further research is required to determine what effect, if any, the ceiling imposed by a texting plan (i.e., the maximum number of texts allowed per month) has on self-reported estimates of numbers of daily texts sent.

Our findings also showed that participants accurately reported the type of input device and its key configuration as well as the orientation of their phone while texting, yet there was far less agreement between the observed and self-reported hands used to hold the phone while texting. This suggests that such cell phone characteristics may be assessed through questionnaires with minimal bias. It is not currently known how the cell phone characteristics may affect the risk of MSDs. However, less keystroke force has been measured with touch screen than with a physical keyboard [28], suggesting greater muscle activation may occur with the latter, which may impact MSD risk. Many more subjects texted in front of the researchers with two hands holding the phone than would be expected given their self-reports. The texting style that we observed during a trial of short duration may not be reflective of the participants’ texting style for extended periods or during naturalistic settings. Hence, these results must be interpreted with caution.

Whether the observed texting task was performed first or last had little impact on participants’ questionnaire responses relative to texting frequency, cell phone device characteristics, or texting styles. Therefore, in future validity studies that employ a similar approach to that used here, either protocol could be used.

Despite the misclassification of texting frequency in this study, the self-report measure likely has utility when a study objective is to rank participants from low to high texting frequency, that is, when a study objective is to determine whether there is any association between a health outcome (e.g., sleep disturbance, academic performance) and daily texting frequency, for example. However, in epidemiologic studies where determining a risk threshold is an objective, we recommend that a less biased measure (i.e., a phone bill) be used to assess the frequency of text messaging.

### Strengths and limitations

One limitation was the small sample size which may have prevented precisely estimating the degree of misestimation of texting frequency by self-report for various daily texting categories. Also, the sample was not randomly recruited. Rather, it was those students who were in the student center at the time of recruitment and who were interested in participating who were enrolled in the study. However, our study population compares favorably with the overall demographics of WVU students, with a slight over-representation of females and African Americans. As we only compared students’ self-reported daily texting frequency with the corresponding measure derived from the previous month’s phone bill, our conclusions are valid only for short-term recall as we did not view previous phone bills for a greater number of months. In addition, because this study is focused on young adults, ages 18 to 24, it is unknown whether the results can be generalized to younger or older populations.

In this study, we elected to use close-ended, categorical response options to obtain self-reported texting frequency. This approach has its advantages and disadvantages compared to an open-ended question format. One of the primary advantages of using open-ended questions is that it allows one to conduct statistical analyses that are not possible with categorical data including calculating intra-class correlation coefficients, for example. However, closed-ended questions can be preferable as they present a lower response burden on the survey taker, lessen survey response time, and are less subject to item non-response, especially in self-administered surveys [29-31]. In using a self-administered survey in this study, the selection of a close-ended question format to obtain self-reported texting frequency made practical sense given these considerations.

As far as we are aware, this is the first study to determine the agreement between self-reported number of daily text messages sent and billing records in young adults, and the first to examine the agreement between self-reported texting styles and phone characteristics and observation data in any population. The findings can be useful in informing future epidemiologic investigations where texting frequency and health outcomes are considered.

## Conclusions

We found subjects to inaccurately report their daily texting frequency and consistent overestimation of texting frequency regardless of self-reported texting volume, in comparison to cell phone billing records. Utilizing a self-reported number of daily texts will be subject to overestimation of exposure. Self-reported texting frequency may be suitable for ranking subjects from low to high in studies where the objective is to determine if there is an association between a health outcome (e.g., sleep disturbance) and daily texting frequency, for example. However, they are not recommended for epidemiologic studies in which determining a risk threshold is an objective. For these studies, it is recommended that a less biased measure, such as a cell phone bill, be used. Further research to investigate the validity of self-reported number of daily text messages sent, phone characteristics, and texting styles is warranted in all age groups.

## Endnotes

^a^We divided the total number of texts in half based on the average ratio (1:1.02) of texts sent to texts received for four subjects who had phone bills that showed the number of messages sent and the number of messages received separately.

^b^Kmax is an empirical measure derived from the marginal totals of the data at hand which are regarded as fixed. Cell frequencies are then modified to reflect the maximum possible agreement given the distribution of the data.
